# The Influence of Polycaprolactone on Structural Changes of Dusts from Molding Sands with Resin-Based Binder before and after the Biodegradation Process

**DOI:** 10.3390/polym14132605

**Published:** 2022-06-27

**Authors:** Katarzyna Major-Gabryś, Iwona Stachurek, Małgorzata Hosadyna-Kondracka, Marta Homa

**Affiliations:** 1 Faculty of Foundry Engineering, AGH University of Science and Technology, Al. Mickiewicza 30, 30-059 Krakow, Poland; 2 Łukasiewicz Research Network—Krakow Institute of Technology, Zakopianska 73, 30-418 Krakow, Poland; iwona.stachurek@kit.lukasiewicz.gov.pl (I.S.); marta.homa@kit.lukasiewicz.gov.pl (M.H.)

**Keywords:** polycaprolactone, resin-based binders, molding sands, biodegradability

## Abstract

Resin-based binders are one of the main materials used in foundry molding and core sands. This work adds to the research on self-curing sands with furfuryl resin, which dominates in the production of large-size castings. The work concerns the possibility of using biodegradable polymers as a component of resin-based binders. Biodegradable or partially biodegradable polymers are a group of materials which have an increasing level of importance in many areas of life and technology. This is mainly due to the increase in waste deposited in landfills, water and soil. This problem also concerns waste from the casting production process with the use of disposable molds with resin-based binders, which are mainly residues from their mechanical regeneration process. The aim of the research presented in this paper was to determine the effect of polycaprolactone (PCL) on the structure of post-regeneration dust from molding sands before and after the biodegradation process carried out in a water environment. Structure studies were carried out by Fourier transform infrared spectroscopy (FTIR) and the changes in the mass by TG/DTA-QMS analysis. The article also presents the results of the research of the morphology carried out by scanning electron microscopy (SEM) and the chemical oxygen demand index (COD) in water extracts of dust samples.

## 1. Introduction

Resin-based binders are one of the main materials used in foundry molding and core sands. This work supplements to the research on self-curing molding sands with furfuryl resin, which dominates in the production of large-size castings. Furfuryl resins and resins modified with furfuryl alcohol used as foundry binders contain 7–90% (practically 30–80%) furfuryl alcohol. The addition of alcohol accelerates the hardening process and increases the bonding capacity, which makes it possible to reduce the addition of binder to the molding sand. Furfuryl resins undergo spatial cross-linking under the influence of heating or chemical agents.

The technology of self-curing molding and core sands with furfuryl resins ensures the attainment of castings with high dimensional accuracy, the ability to prepare complex forms, bonding at ambient temperature, an easy process of making molds, good knockout properties and the ability to mechanical regeneration of the used molding sands. The disadvantages of this technology include, first of all, the high harmfulness of gases emitted, mainly when pouring with liquid melt, and the problem with the disposal of post-regeneration waste from the applied molding sands.

Post-production waste from used molding and core sands is the main group of waste generated by foundry industry. It is assumed that, on average, one ton of castings generates 0.6–1.0 ton of used sand [1,2,3]. The world production of castings at the level of 100 million tons, including iron castings in chemically hardened molding sands in the amount of 30 million tons, and assuming a degree of regeneration at the level of 40–50%, gives 15–18 million tons of the used sand [1,2,3].

This problem is particularly important as most of the commonly used raw materials for their production are not decomposable or are difficult to decompose. These materials are resistant to numerous environmental factors; most of them are not damaged by water, air, sunlight or the action of microorganisms. Degradation is defined as an irreversible, often multi-step process leading to marked changes in chemical structure, usually resulting in a loss of performance in the material. The degradation can be caused by various factors in the environment. When the degradation takes place under the influence of electromagnetic radiation, mechanical forces, heat, and active chemical compounds, it is defined as abiotic degradation. When the degradation process is caused by the action of biological factors (living organisms), mainly enzymes produced by various microorganisms, i.e., bacteria or fungi, it is a biotic degradation (biodegradation, or biological degradation). The potential biodegradability of a material, which has been established or is predicted on the basis of appropriate laboratory tests under strictly defined, controlled conditions, is called specific biodegradability. In turn, total (final) biodegradability is the total decomposition of the material by microorganisms, either in the presence of oxygen (aerobic biodegradation) to CO_2_, H_2_O, mineral salts with the formation of new biomass, or under anaerobic conditions (anaerobic biodegradation, biomethanization) to CO_2_, CH_4_, inorganic salts and biomass [4,5,6].

The growing interest in photodegradable and biodegradable materials with a controlled lifetime, in which their degradation should start only after fulfilling their task, also includes materials for obtaining molds for large-size castings. Therefore, another current direction of research and development in the field of foundry molding materials is the possibility of obtaining biodegradable, environmentally friendly materials.

Works on this issue began in the 1990s [7,8,9] and has continued until now.

In previous studies, K. Major-Gabryś proposed a new approach to the issue consisting of the use of biodegradable polymer (polycaprolactone) as a component of resin-based binders available on the market. Such a solution will reduce the number of harmful residues after mechanical regeneration of molding sands and accelerate the biodegradation process of the synthetic binder [3,10,11,12,13,14,15,16]. The choice of PCL as a biodegradable component was dictated by the fact that, according to the literature data [17,18,19,20,21], this polymer is partially compatible or mechanically compatible with some polymers, such as polyvinyl acetate (PVAc), polystyrene (PS), polycarbonate, polyvinyl chloride (PVC), styrene-acrylonitrile copolymer (SAN), poly (hydroxyether), etc. This feature of polycaprolactone enables the formation of various “bio-destructive” mixtures using it as a biodegradable component [22]. Initial studies of “bio-destructive” polymer blends using PCL as a biodegradable component relate to a PC/polyolefin blend system based on polyolefins, such as LDPE (low density polyethylene) and PP (polypropylene). More extensive studies on the biodegradability of PCL/polyolefin blends, including the relationship between biodegradability and phase structure, have been presented by Iwamoto and Tokiwa [23]. Polycaprolactone is also used as a plasticizer in blends with aliphatic polyesters, cellulose esters, aromatic polyesters, polycarbonates, styrene-containing polymers (e.g., polystyrene), polyolefins, block copolymers and Novolak resin [24]. The use of polycaprolactone as a plasticizer in molding and core sands is also very important but is not the subject of this paper. The presentation of the above examples is intended to show the versatility of the use of polycaprolactone as a component of various materials.

Due to the fact that residues of post-regeneration materials are an unresolved problem from the point of view of environmental protection, taking up the topic of adding biodegradable material in order to reduce the amount of harmful waste and accelerate its biodegradation seems fully justified.

The article presents the results of research on the influence of polycaprolactone (PCL) on the structure of post-regeneration dust from molding sands before and after the biodegradation process. Dust after regeneration of standard molding sand with resin-based binders and dust after the sand regeneration process with the addition of biodegradable material (PCL) were tested. The biodegradation process was carried out in an environment reflecting real environmental conditions. The structure was determined by Fourier transform infrared (FTIR) spectroscopy. The paper also presents preliminary studies of the morphology and grain size by scanning electron microscopy (SEM) and the results of determination of the chemical oxygen demand index (COD) of aqueous extracts of dust samples after the (bio) degradation process.

## 2. Materials and Methods

### 2.1. Materials

The following materials were selected as components for molding sands that were subjected to mechanical regeneration:

- Furfuryl resin, without the presence of nitrogen; free formaldehyde in the range of 0.05–0.15%; the amount of furfuryl alcohol 78%;
- Hardener—an aqueous solution of paratoluenesulfonic acid;
- Polycaprolactone (PCL)—a biodegradable additive and plasticizer with the formula –(O(CH2)5CO)-_n_; molecular weight 50,000 Da and melting point 58–60 °C; a biodegradable polymer in solid form with an end of hydroxyl group from Polysciences Inc., Warrington, PA, USA.

The dust samples after the process of mechanical regeneration of molding sands: standard molding sand with organic binders (MS no. 1) and molding sand with organic binder and the addition of polycaprolactone PCL (MS no. 2) were the subjects of our examination. The composition of the molding sands and dust-sample types are presented in Table 1.

The material has been processed the biodegradation process. Three g samples of dust were placed in polypropylene containers and filled 100 mL of water from the Vistula River, with a confirmed composition and specific physicochemical properties (Table 2). The samples were stored in closed containers at room temperature for 3 months.

### 2.2. Methods

Structure studies were carried out by Fourier transform infrared spectrometry (FTIR) using Digilab Excalibur FTS 3000 (Bio Rad, Hercules, CA, USA) spectrometer with a standard DTGS detector. The test specimens were obtained at room temperature by the KBr pelletization method. The number of scans was the same for all samples, the results were recorded in the wavenumber range from 4000 to 400 cm^−1^ with a resolution of 4 cm^−1^.

Selected samples were subjected to the thermogravimetric test and the differential thermal analysis (TG/DTA) in order to determine the changes in the mass of the tested sample and the thermal effects related to the changes in the sample (physical, chemical). The TG/DTA-QMS measurements were carried out using the STA 449 F3 Jupiter^®^ thermal analyzer coupled to QMS 403C Aëolos by Netzsch (Selb, Germany) equipped with a TG-DTA sensor. The test was proceeded under the following conditions: pressure 10^−2^ mbar; inert gas Ar (99.9992%) 70 mL/min; heating speed 15 K/min. Measurements were determined using the Netzsch (Selb, Germany) Proteus Thermal Analysis 6.1.0 software. The residual gas analysis was carried out on the basis of the literature data from [26].

The determination of the morphology and chemical composition by X-ray microanalysis in selected microareas of the dust samples before and after the biodegradation process was performed using the Jeol JSM 6460LV (Tokyo, Japan) scanning electron microscope (SEM) with the INCA X- EDS-act Energy 350 by Oxford Instruments (Abingdon, UK) spectrometer. The powder samples were attached to a carbon tape and vaporized with platinum.

**Figure 1:**
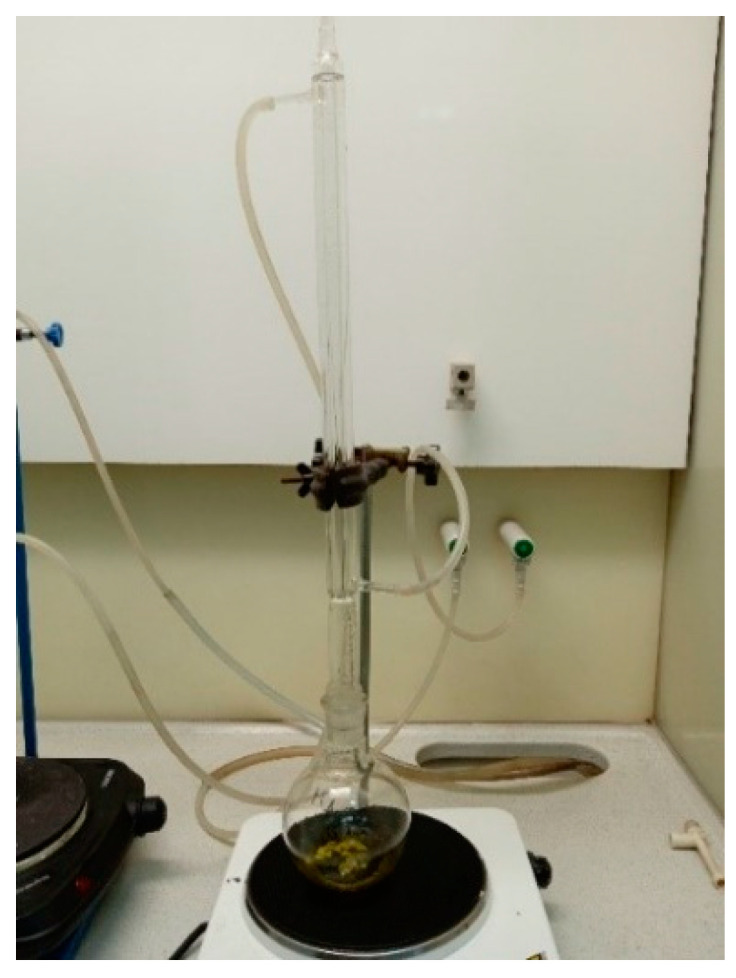
Heating the mixture in the reaction flask.

The determination of the COD of the water extracts after the biodegradation process of dust samples from molding sand MS no. 1 and MS no. 2 was carried out in accordance with the PN-ISO 6060:2006 standard “Water quality. Determination of the chemical oxygen demand” [27]. In the first stage, 10 cm^3^ of the aqueous extract were collected into the reaction flask, to which 5 cm^3^ of the potassium dichromate solution and a few porous glass spheres were added. After thorough mixing, 15 cm^3^ of a solution of silver sulphate in sulfuric acid were added very slowly to the solution. The reaction flask was then connected to a reflux condenser and the reaction mixture was heated to reflux and then held at 148 °C for 110 min. At this time, the reaction mixture was cooled rapidly to about 60 °C, diluted to about 75 cm^3^, and then cooled to room temperature. The course of the first stage of the determination-heating of the mixture in the reaction flask is shown in Figure 1.

The excess of potassium dichromate (VI) was titrated with ammonium and iron (II) sulfate. Ferroin was used as an indicator [27]. Chemical oxygen demand was calculated from the formula (Equation (1)):

(1) $$X_{COD}=\frac{8000\times c\times \left(V_{1}-{\;V}_{2}\right)}{V_{0}}$$

where:

c—concentration of ammonium and iron (II) sulphate (VI) expressed in mol/dm^3^

V_0_—the volume of the analytical sample expressed in cm^3^

V_1_—volume of ammonium and iron (II) sulphate (VI) used for titration in the blank sample, expressed in cm^3^

V_2_—volume of ammonium and iron (II) sulphate (VI) used to titrate the analytical sample, expressed in cm^3^

8000—molar mass ½ O_2_ expressed in mg/dm^3^

## 3. Results

### 3.1. Structure Analysis by FTIR Spectroscopy

#### 3.1.1. Determination of the Structure of Dust Samples before the Biodegradation Process

**Figure 2:**
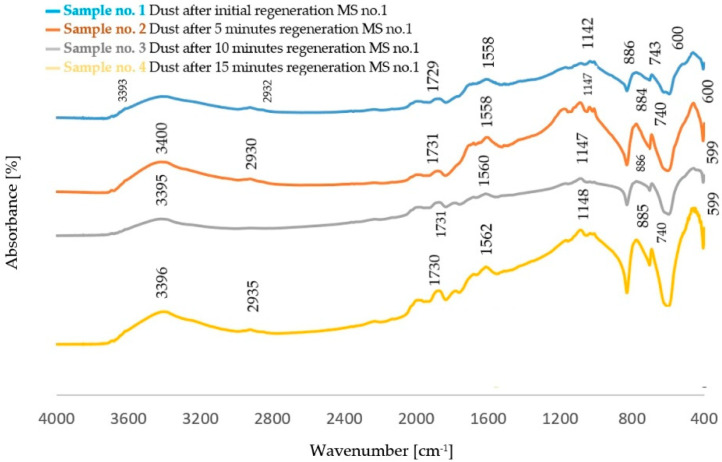
FTIR spectrum obtained for dust samples after the regeneration process of molding sand MS no. 1 before the biodegradation process. Absorbance [%] Wavenumber [cm^−1^].

**Figure 3:**
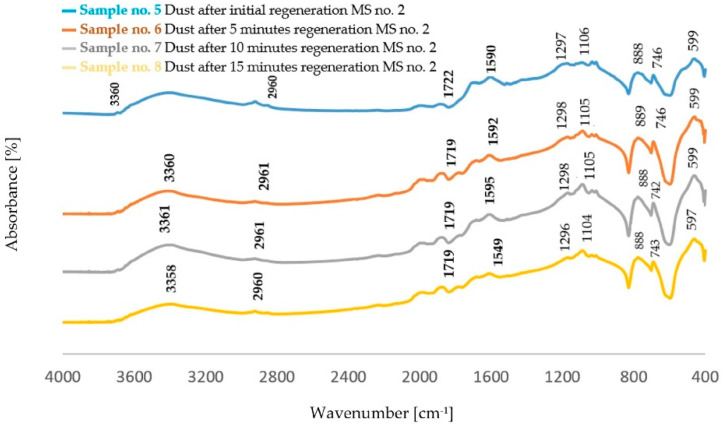
FTIR spectrum obtained for dust samples after the regeneration process of molding sand MS no. 2 before the biodegradation process.

The FTIR spectra obtained for dust samples after the regeneration of molding sand are presented in Figure 2 and Figure 3. Table 3 shows the identification of the absorption bands determined for furfuryl resin, in Table 4 the bands characteristic for polycaprolactone obtained on the spectra from dust samples of molding sand MS no. 2 after the regeneration of the molding sand with the addition of a biodegradable component.

In the spectra obtained for all the dust samples obtained after the regeneration of the standard molding sand (dust from MS no. 1) and the molding sand with a 5% addition to the resin of a biodegradable component (dust from MS no. 2), the most characteristic is the band originating from the binder characteristic for phenol-furfuryl resin. These vibrations occur in the area of 1149–1142 cm^−1^ [28]. In the 3400 cm^−1^ region, there is a band corresponding to the stretching vibration of the O-H bond [29]. Another characteristic band is the one corresponding to the deformation vibrations of C-H bonds characteristic for the furan ring, occurring around 885 cm^−1^ [30]. In the region of 1360 cm^−1^, on the other hand, OH- group bands are visible, which correspond to vibrations originating from the furan or phenol ring [29]. These bands are characterized by a low intensity. The spectra obtained for post-regeneration dust from the molding sand with the addition of PCL also show bands characteristic of aliphatic polyesters [31,32].

#### 3.1.2. Determination of the Structure of Dust Samples after the Biodegradation Process

**Figure 4:**
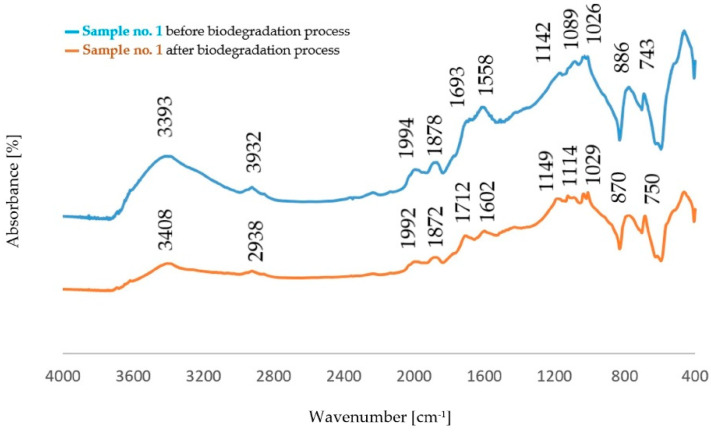
Comparison of the absorption bands for dust samples of molding sand MS no. 1 after initial regeneration, before and after the biodegradation process.

**Figure 5:**
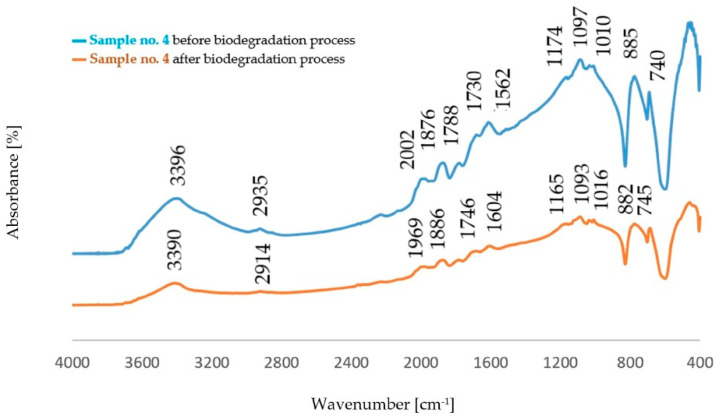
Comparison of the absorption bands for dust samples of molding sand MS no. 1 after 15 min of mechanical regeneration process, before and after the biodegradation process.

**Figure 6:**
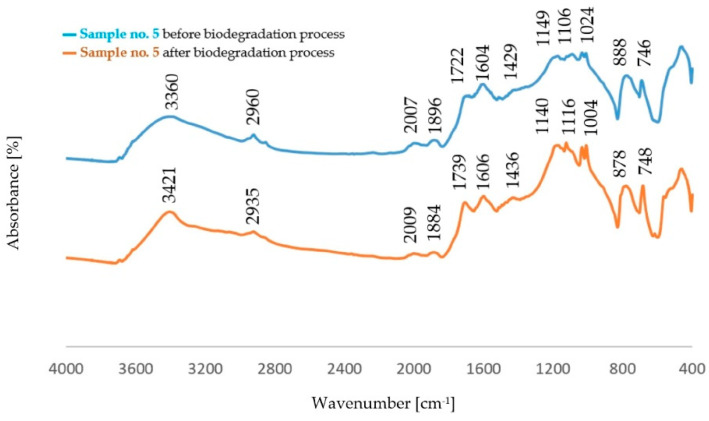
Comparison of the absorption bands for dust samples of molding sand MS no. 2 after initial regeneration, before and after the biodegradation process.

**Figure 7:**
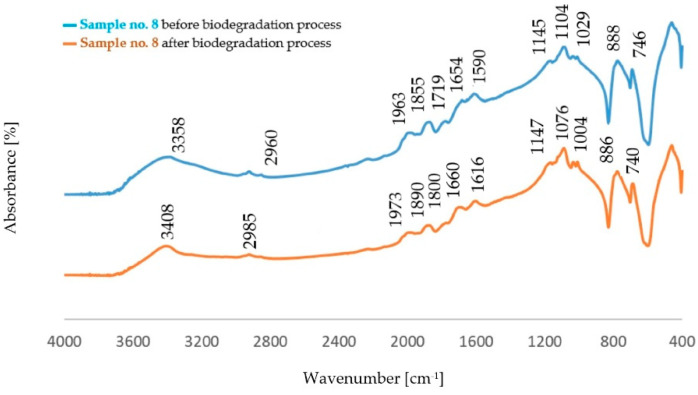
Comparison of the absorption bands for dust samples of molding sand MS no. 2 after 15 min of mechanical regeneration process, before and after the biodegradation process.

The comparison of the absorption bands for dust samples before and after the biodegradation process is shown in Figure 4, Figure 5, Figure 6 and Figure 7.

The analysis of FTIR spectra of the dust samples after the regeneration process and after the biodegradation process showed slight shifts of a small number of absorption bands towards lower wavenumber values. On the other hand, slight changes were visible in the intensity of some absorption bands. In all spectra, an increased intensity of the band originating from the stretching vibration of the OH− (ν_O-H_) groups was found [29]. The spectra obtained for the dust samples from the standard molding sand MS no. 1 showed a decrease in the intensity of the band in the area of 2942 cm^−1^ coming from the C−H group. In the case of dust after the regeneration process of the molding sand with the addition of a biodegradable component, an increase in the intensity of the band originating from the C−H group was observed in this area. An increase in the band intensity in the area of 1142 cm^−1^ and in the area of 1730 cm^−1^ was found for dust samples obtained from the standard molding sand MS no. 1. These bands correspond to vibrations originating from the bond characteristic for the resin and from the C=O group, respectively. For these samples, an increase in the intensity of the band in the region of 885 cm^−1^ derived from the C-H group corresponding to the deformation vibration occurring in the furan ring was also found [30,33,34,35]. For samples of dust sediments obtained after the regeneration process with the addition of a biodegradable component, the study of the sediment structure after the biodegradation process also showed a decrease in the intensity of the band resulting from the binding of ν_C=O_ characteristic for aliphatic polyesters [31,32,33,34,35,36].

### 3.2. The Changes in the Mass by TG/DTA-QMS Analysis

**Figure 8:**
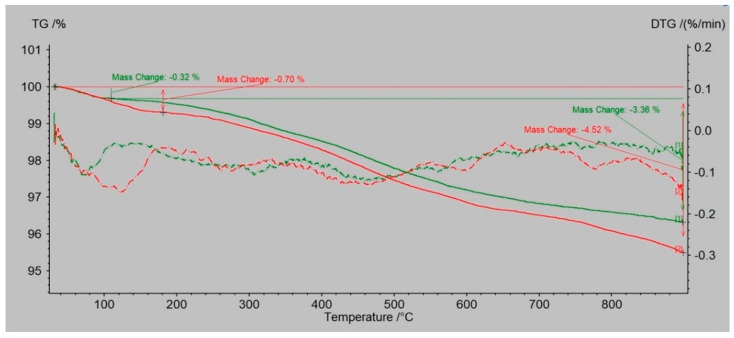
TG/DTG curves obtained by heating the sample no. 1—the dust sample from MS no. 1 after initial regeneration; before biodegradation (green line) and after biodegradation (red line).

**Figure 9:**
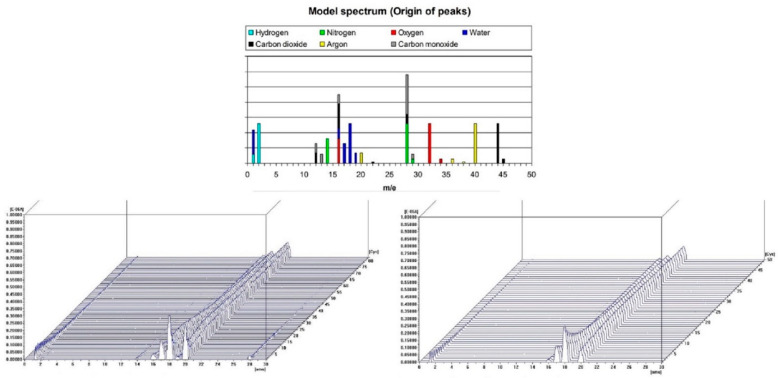
Changes in the chemical composition of residual gases during TG/DTA tests of the sample no. 1—the dust sample from MS no. 1 after initial regeneration; before biodegradation (on the left) and after biodegradation (on the right). On top an interpretation of the QMS spectrum based on [26] is presented.

**Figure 10:**
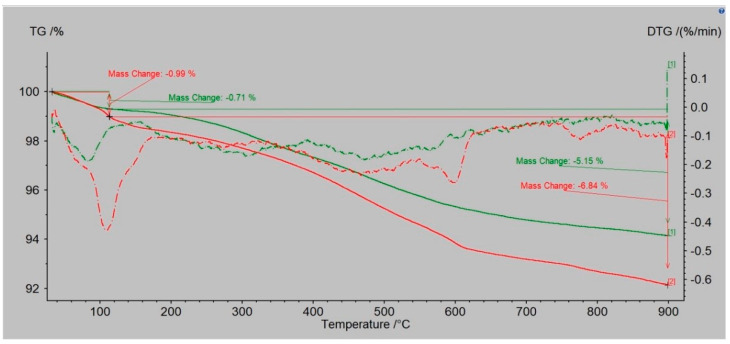
TG/DTG curves obtained by heating the sample no. 5—the dust sample from MS no. 2 after initial regeneration; before biodegradation (green line) and after biodegradation (red line).

**Figure 11:**
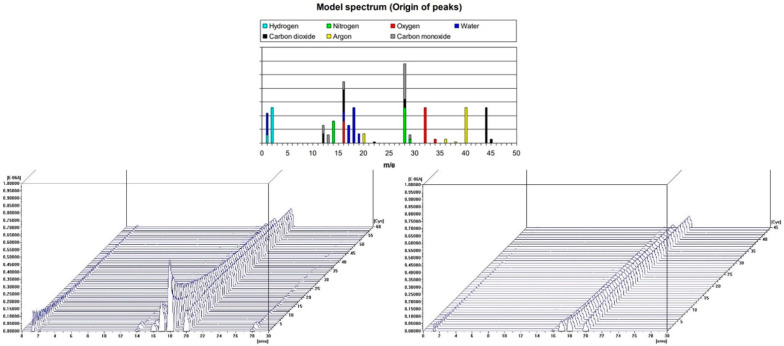
Changes in the chemical composition of residual gases during TG/DTA tests of the sample no. 5—the dust sample from MS no. 2 after initial regeneration; before biodegradation (on the left) and after biodegradation (on the right). On top an interpretation of the QMS spectrum based on [26] is presented.

Dust samples from the molding sand MS no. 1 and MS no. 2, before and after the biodegradation process were selected to show the changes occurring in them as a result of heating the temperature up to 900 °C. Figure 8 and Figure 9 present results for dust samples from molding sand MS no. 1 after initial regeneration (Sample 1) before and after the biodegradation process. Figure 10 and Figure 11 presents results for dust samples from molding sand MS no. 2 after initial regeneration (Sample 5) before and after the biodegradation process.

The total mass change for Sample no. 1 up to 900 °C before biodegradation process was 3.36% and for the same sample after biodegradation process was 4.52% (Figure 8). The QMS spectrum presented on the Figure 9 shows selected values of atomic masses (AMU) that correspond with chemical compounds:

1-H^+^; 2-H^2+^, He^+^; 14-N; 16-O^+^, CH4^+^, CO_2_, CO; 17-OH^+^; 18-H_2_O^+^; 19-H_2_O^+^; 20—Ar (sample before biodegradation) and 1-H^+^; 2-H^2+^, He^+^; 16-O^+^, CH4^+^, CO_2_, CO; 17-OH^+^; 18-H_2_O^+^; 20—Ar (sample after biodegradation).

The total mass change for Sample no. 5 up to 900°C before biodegradation process was 5.15% and for the same sample after biodegradation process was 6.84% (Figure 10). The QMS spectrum presented on the Figure 11 shows selected values of atomic masses (AMU) that correspond with chemical compounds:

1-H^+^; 2-H^2+^, He^+^; 16-O^+^, CH4^+^, CO_2_, CO; 17-OH^+^; 18-H_2_O^+^; 20—Ar (sample before biodegradation) and 1-H^+^; 2-H^2+^, He^+^; 16-O^+^, CH4^+^, CO_2_, CO; 17-OH^+^; 18-H_2_O^+^; 20—Ar (sample after biodegradation).

### 3.3. Microscopic Observations (SEM, EDS)

**Figure 12:**
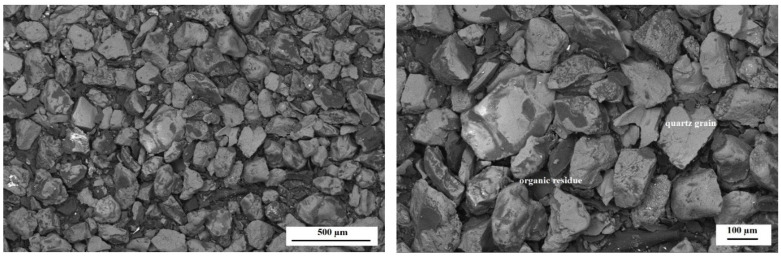
The morphology of the microstructure of the sample no. 1—the dust sample from MS no. 1 after initial regeneration; SEM magnification 50× (left) and 100× (right). The structure of the sample with quartz grains and residues of organic material.

**Figure 13:**
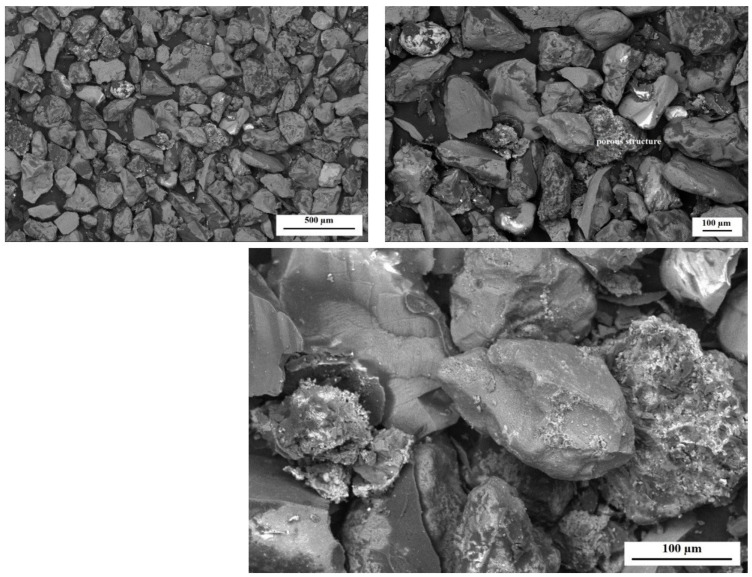
The morphology of the microstructure of the sample no. 1—the dust sample of MS no. 1 after initial regeneration and biodegradation process; SEM, magnification 50× (left), 100× (right) and 250× (at the bottom). Quartz grains and porous structures are visible.

**Figure 14:**
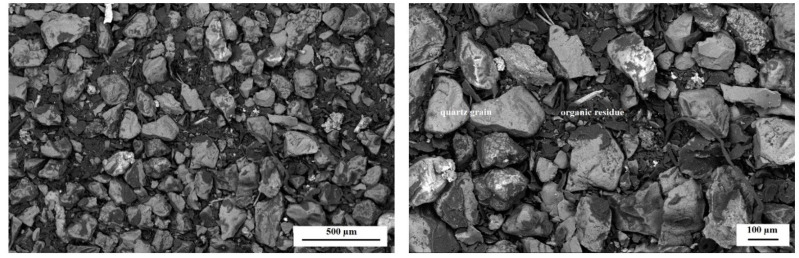
The morphology of the microstructure of the sample no. 5—the dust sample from MS no. 2 after initial regeneration; SEM, magnification 50× (left) and 100× (right). The structure of the sample with quartz grains and large amounts of organic residues.

**Figure 15:**
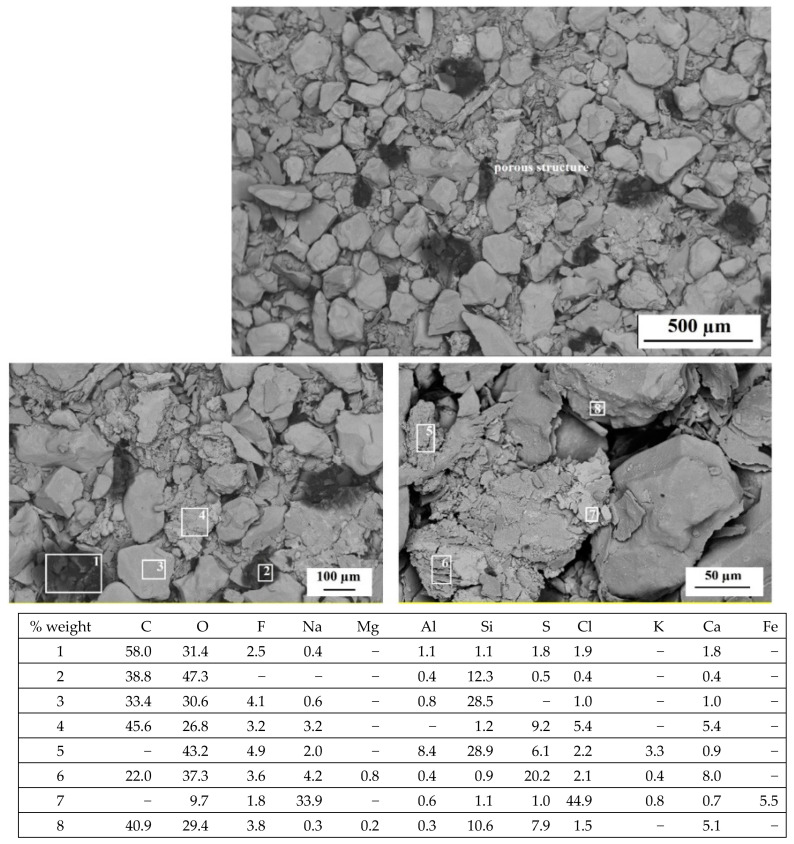
The morphology of the microstructure of the sample no. 5—the dust sample of MS no. 2 after initial regeneration and biodegradation process with chemical composition in selected microareas 1 to 8; SEM, magnification 50× (on top), 100× (left) and 350× (right) with EDS analysis (table at the bottom). Quartz grains and porous structures are visible.

The morphology studies were carried out in order to determine the changes that could have occurred under the influence of the environmental factor during the biodegradation process. Figure 12 and Figure 13 show SEM micrographs obtained for dust samples from molding sand MS no. 1 after regeneration before and after the biodegradation process. Figure 14 and Figure 15 show SEM micrographs obtained for the samples from molding sand MS no. 2 after regeneration before and after the biodegradation process.

For comparative purposes, observations of dust samples for both molding sands MS no. 1 and MS no. 2 after initial regeneration were present. In the case of sample no. 1 (Figure 12), it can be observed that in its structure there are grains of the matrix (quartz sand); on the surface of and between these grains, there is an organic residue from the binder (dark color). After the biodegradation process, the morphology of the sample slightly changed and on the surface of some grains and in the place of bonding bridges (Figure 13), the formation of a new porous structure can be observed.

In the case of sample no. 5 derived from the molding sand MS no. 2, in which a biodegradable agent was applied to the binder, it can be observed that, as a result of the regeneration process, a much greater amount of organic residue was obtained than in the case of the sample no. 1 from MS no. 1 (Figure 12 and Figure 14). There are also matrix grains in the structure (Figure 14). After the biodegradation process in the aquatic environment in the sample no. 5 numerous porous structures appear, mainly between the grains of the quartz matrix (Figure 15). EDS analysis of these products confirmed the presence of mainly C and O and significant amounts of Si in areas 2, 3, 5, 8 (Figure 15). In area 7 the presence of a large amount of Cl and Na was identified. Analyses in selected areas also confirmed the presence of elements, such as F, Mg, Al, S, K, Ca and Fe. C, O and S are most likely derived from resin binder and sulfur hardener. Oxygen can also come from the grain matrix in combination with Si, and sulfur can also come from the water used in the biodegradation process, as well as Cl, Na, F, Mg, K and Ca (Table 2 and [36]).

### 3.4. COD Measurement

The scope and speed of biodegradation transformations, as it has already been presented in the theoretical part, is conditioned by a number of factors that should characterize the environment in which the process is carried out. In addition to factors, such as the content of nutrients, temperature and pH, the access of oxygen plays an important role. Chemical oxygen demand (COD) is an indicator that determines the amount of oxygen necessary for the oxidation of organic and inorganic compounds contained in water. Laboratory methods determine it by determining the amount of oxygen taken from oxidants (potassium dichromate (VI) K_2_Cr_2_O_7_, potassium periodate (KJO_3_, potassium permanganate KMnO_4_) that is needed to oxidize these compounds to the highest degree of oxidation under given conditions [27].

The results of the chemical oxygen demand determination are presented in Table 5.

The data presented in Table 5 show that for the dust from the standard molding sand (MS no. 1), the highest chemical oxygen demand occurs for the dust sample collected after initial regeneration (sample no. 1) and amounted to 691 mg/dm^3^. The COD value then decreases with the regeneration time and remains constant after 10 min of the regeneration process. The addition of a biodegradable component (MS no. 2) to the molding sand will significantly affect the value of chemical oxygen demand. For sample 5, that is a dust sample taken directly after the initial regeneration process, the determined COD value was 1152 mg/dm^3^. This is consistent with the conducted microscopic observations; sample no. 5 is characterized by an increased amount of organic residue in relation to the sample no. 1 and therefore the COD value is much higher. As in the case of the dust from the standard molding sand (MS no. 1), the index value decreased with the duration of the regeneration process and after 15 min, it amounted to 413 mg/dm^3^.

## 4. Discussion

The aim of the research was to determine the effect of polycaprolactone on the structural changes in dust from molding sand with a resin-based binder before and after the biodegradation process in a water environment. The tests were carried out on dust samples after the process of mechanical regeneration of molding sands with a resin-based binder of a standard composition and with the addition of biodegradable material. The analysis of FTIR spectra carried out for the original dust samples showed that the addition of polycaprolactone in the amount of 5% to the furfuryl resin causes the appearance of a band at the wavenumber of 1104 cm^–1^ corresponding to the vibration of the CH_2_ group in PCL. The analysis of FTIR spectra also showed a slight increase in the band intensity at the wavenumber of 1720 cm^–1^ caused by the C=O stretching vibration of the carbonyl group characteristic for aliphatic esters. However, no significant changes were found in the dust structure after the biodegradation process in relation to the initial dust samples. Research has shown that, first of all, the intensity of some absorption bands changes, while their positions and shapes remain practically unchanged. In all spectra, an increased intensity of the band originating from the stretching vibration of the OH− (ν_O-H_) groups was found, which may indicate a partial degradation of the structure, mainly PCL. On the other hand, fragments of biodegradable chains migrate to the incubation solution.

For both molding sands MS no. 1 and 2, the weight loss of the dust sample before biodegradation was lower (3.36% for MS no. 1 and 5.15% for MS no. 2) than after the biodegradation process (4.52% for MS no. 1 and 6.84% for MS no. 2)—the differences are appropriately on the levels 1.16% (MS no. 1) and 1.69% (MS no. 2). Higher losses of total mass up to 900 °C were recorded for MS no. 2 with the PCL addition. QMS analysis confirmed the presence of gaseous components that are normal for this type of molding sand with an organic binder.

Scanning electron microscopy examinations showed that dust sample no. 5 from molding sand MS no. 2 (with the addition of PCL in the binder) after initial regeneration had a larger amount of organic residue than dust sample no. 1 from molding sand MS no. 1 (without the addition of PCL) after initial regeneration. The analysis of the local chemical composition in the micro-areas showed the presence of the following elements: silicon (Si), oxygen (O) and carbon (C) as the main components of the molding sand based on quartz sand. Irregular, porous structures observed on the photomicrographs obtained for dust samples after the regeneration process, mainly of the molding sand with the addition of a biodegradable component, indicate the presence of Cl, Na, F, Mg, K and Ca as well, characteristic elements of river water [37]. The sulfur could come from the molding sand hardener or from river water. 

The conducted measurements of the determination of the chemical oxygen demand index showed a decrease in the COD value along with the duration of the mechanical regeneration process for all tested water extracts. The decrease in the index value is the result of the decreasing number of organic substances in the water extract sample, which can oxidize. The tests were carried out in closed systems; the samples were placed in water of known chemical composition. The presence of chlorides at the level of 369 mg/L was found in water and it could act as an oxidant in the tested environment. The highest COD value was reported for the dust sample no. 5 from the molding sand MS no. 2 with the addition of a biodegradable component after the initial regeneration and biodegradation process. This is consistent with the results of the microscopy observations about the amount of organic residue. The COD value for the sample no. 5 resulted in a faster oxidation of organic compounds. At the same time, the obtained results indicate no influence of polycaprolactone on the organic substance of the molding sand, including the process of its decomposition into inorganic compounds. This would be confirmed by the lower COD values.

## 5. Conclusions

The conducted research of the biodegradation process in a water environment and the analysis of the obtained results allowed for the formulation of the following conclusions:

- It is possible to use biodegradable polymer as a component of foundry resin-based binder.
- There were no significant changes in the dust samples’ structures before and after the biodegradation process. An increased intensity of the band originating from stretching vibrations of the OH− groups (ν_O-H_) was found. This effect may confirm the partial degradation of the structure, mainly PCL.
- Dust samples after the biodegradation process had higher weight loss than the same samples before biodegradation. Dust samples from molding sand with PCL addition had higher weight loss than dust samples from molding sand without PCL addition.
- Morphology studies showed the presence of irregular, porous structures mainly in the dust samples from molding sand MS no. 2 after the biodegradation process.
- It was found that increasing the regeneration time reduces the amount of organic matter in the tested samples, which has a significant impact on the decrease in the COD value along with the duration of the process.
- The conducted research and the obtained results indicate the directions of further research and work in the selection of components of molding sand with biodegradable properties, which should be carried out in terms of determining the minimum and maximum amount of polycaprolactone as an addition of a biodegradable component to the molding sand. It would be also essential to carry out a biodegradation process in longer time - between 6 and 12 months. Important for determining the properties of biodegradable materials is the biodegradation process in which the inoculum should be activated sludge used in sewage treatment plants or under the influence of microorganisms that break down aromatic hydrocarbons, e.g., bacteria such as *Aeromonas*, *Pseudomonas, Flavobacterium* and *Bacillus*.
- The next step of research would be a structural and molecular weight analysis of the polymer.

## Figures and Tables

**Table 1:** Designation of dust samples and composition of molding sands used for mechanical regeneration.

Sample No.	Designation of Samples	Molding Sand’s Composition
1	Dust after initial regeneration	*Molding sand MS no. 1* silica sand 100 p.b.w.; furfuryl resin 1.1 p.b.w. hardener 0.55 p.b.w.
2	Dust after mechanical regeneration: 5 min	
3	Dust after mechanical regeneration: 10 min	
4	Dust after mechanical regeneration: 15 min	
5	Dust after initial regeneration	*Molding sand MS no. 2* silica sand 100 p.b.w.; furfuryl resin 1.045 p.b.w. (95%) PCL 0.055 p.b.w. (5%); hardener 0.55 p.b.w.
6	Dust after mechanical regeneration: 5 min	
7	Dust after mechanical regeneration: 10 min	
8	Dust after mechanical regeneration: 15 min	

**Table 2:** Main compounds and elements with selected specific physicochemical properties of water from the Vistula River used for biodegradation test [25].

Tested Feature	Unit	Result
pH	-	7.6 (in 22.4 °C)
specific electrical conductivity	µS/cm	1568 (in 25 °C)
chlorides	mg/L	369
sulfur	mg/L	65
nitrates	mg/L	5.7
Zn	mg/L	0.07
Pb	mg/L	0.024
Cu	mg/L	0.004
Ni	mg/L	0.0025
chemical oxygen demand	mg/L	20
dissolved oxygen	mg/L	7.6

**Table 3:** Absorption bands determined for the resin [28,29,30,31,32].

Group	Wavenumber (cm^−1^)	
	Dust from MS no. 1	Dust from MS no. 2
O-H stretch vibration ν_O-H_	3400–3393	3361–3358
C-H	2935–2930	2961–2958
C=O	1731–1729	1768–1762
C=C	1562–1558	1595–1549
OH vibrations from the furan or phenolic ring (OH group), deforming the bond along the plane	1361–1357	1350–1345
vibration from the bond characteristic of phenol-furfuryl resin	1148–1142	1149–1146
C−H deforming vibrations from furan ring	886–884	889–887
skeletal oscillation from bonds found in furfuryl alcohol	743–740	746–742
vibration from the furan ring	600–599	599–597

**Table 4:** Bands determined for polycaprolactone [28,29,30,31,32].

Group	Wavenumber (cm^−1^)
O-H stretch vibration ν_O-H_	3361–3358
CH_2_-O asymmetric stretching vibration ν_O-H_	2946–2944
CH_2_—symmetrical stretching vibration ν_C-H_	2865–2859
CO_2_—stretching vibrations ν_C = O_ from aliphatic esters	1722–1719
C-H asymmetric stretching vibration ν_C-H_	1471–1439
CH_2_—asymmetric stretching vibration ν_C-H_	1369–1367
C−O; C−C; crystal phase stretching vibrations from _cr_ν_C=O_ and _cr_ν_C-C_	1298–1296
C−O−C asymmetric vibration ν_C-O-C_	1241–1240
CH_2_	1106–1104
C−H asymmetric stretching vibration ν_C-H_	731–730

**Table 5:** COD value determined for water extracts of dust samples after the biodegradation process.

Sample Number	Determined COD Value; (mg O_2_/dm^3^)
1	691
2	586
3	442
4	446
5	1152
6	691
7	516
8	413

## Data Availability

The data presented in this study are available on request from the corresponding author.
